# Supplementary material for: A novel C. elegans respirometry assay using low-cost optical oxygen sensors
Source: bioRxiv. 2025 May 19:2025.05.16.654527. Preprint. [Version 1] doi: 10.1101/2025.05.16.654527 (PMC12258713; doi:10.1101/2025.05.16.654527)
Supplement: Supplement 1 [file NIHPP2025.05.16.654527v1-supplement-1.pdf]

bioRxiv preprint doi: https://doi.org/10.1101/2025.05.16.654527; this version posted May 19, 2025. The copyright holder for this preprint (which was not certified by peer review) is the author/funder, who has granted bioRxiv a license to display the preprint in perpetuity. It is made available under aCC-BY 4.0 International license.

Supplementary Information

OCR protocol script

All OCR assays were conducted using the time-resolved fluorescence protocol described in Table 2 of the main text with the following BMG FLUOstar script:

BMG FLUOstar OCR script:

```
st1: = "OxoPlate protocol" #Pre-configured OxoPlate protocol

NumberOfReadings: = n

wait for 10 min

for i=1 to NumberOfReadings do begin

        ID1:="Reading " i

        R_run "<st1>"

 If i = 3 Then begin

        Ask "Add drug A and click Yes to continue " ("Continue")

 End

 If i = 6 Then begin

        Ask "Add drug B and click Yes to continue " ("Continue")

        End

Next i
```

Pauses were implemented every 3 cycles to allow for the plate to be ejected from the reader and treated with respiratory inhibitors. The initial wait period (10 minutes) was included to ensure the temperature of the wells had equilibrated with the internal temperature of the plate reader, and was estimated by examining the stabilisation of oxygen levels in solutions of Milli-Q water following a shift from 20 °C (laboratory temperature) to 25 °C (the internal temperature of the plate reader; Figure S4), which occurred within approximately 10 minutes of measurement. For each assay, the NumberOfReadings (n) was changed as necessary. Sample size analysis was

bioRxiv preprint doi: https://doi.org/10.1101/2025.05.16.654527; this version posted May 19, 2025. The copyright holder for this preprint (which was not certified by peer review) is the author/funder, who has granted bioRxiv a license to display the preprint in perpetuity. It is made available under aCC-BY 4.0 International license.

performed with n = 2; dose-response analysis with n = 6, and sequential drug addition experiments with n = 9.

Automated OCR calculation script

To expedite the calculation of OCRs from internally referenced (I_R) sensor responses, we created a set of functions in R v. 4.3.3 to automatically calculate oxygen concentrations, fit linear regressions within user-defined measurement windows, extract the regression coefficient, multiply the result by -1, and normalise to worm number (or any alternative normalisation parameter). This script requires that the data be formatted with a single "time" column (which must be in lowercase and formatted in seconds), and multiple I_R data columns with the name of the condition specified first, followed by an underscore and a designation for the replicate (see Figure S5 for an example data sheet). This script also requires pre-determined calibration constants derived from oxygen-free (k_0) and oxygen-saturated (k_{100}) calibration solutions (see OxoPlate sensor calibration), and the pre-installation of the *tidyverse* and *forstringr* packages. For the OCR calculation script, the OCR calculation window is set by the interval value (the number of measurements for regression-fitting), and the total length of each kinetic window (as set by the number of cycles in the BMG FLUOstar protocol) is set by the step value.

[O_2] calculation function:

```
k0 <- #User-defined k0 value

k100 <- #User-defined k100 value

o2.fun <- function(data, k0, k100){

 df <- cbind(data[,1], 100*(k0/data[,-1]-1)/(k0/k100-1))

 return(df)

}
```

Regression-fitting and differentiation function

```
get_slope <- function(x, y) {

 lm_model <- lm(y ~ x) #Fits linear regression

 return(coef(lm_model)[2]) #Extracts regression coefficient

}
```

bioRxiv preprint doi: https://doi.org/10.1101/2025.05.16.654527; this version posted May 19, 2025. The copyright holder for this preprint (which was not certified by peer review) is the author/funder, who has granted bioRxiv a license to display the preprint in perpetuity. It is made available under aCC-BY 4.0 International license.

OCR function:

```
OCR.fun <- function(data, interval, step, worm.no) {


 slopes <- numeric() #Initialises empty vector to store OCRs for each kinetic window

 col_names <- character() #Initialises empty vector to store sample name information

 interval_numbers <- numeric() #Initialises empty vector to record the kinetic window

 interval_number <- 1 #Sets kinetic window number to 1 prior to running the loops

 for (i in seq(1, nrow(data), by = step)) { #Loop to subset the data for each kinetic window

  subset_data <- as.data.frame(data[i:(i + interval - 1), ])

  time_subset <- subset_data$time #Extracts time column for calculating OCRs

  for (col in 2:ncol(subset_data)) { #Loop to fit regressions to each sample within each window

   slope <- get_slope(time_subset, subset_data[, col])

   slopes <- c(slopes, slope / worm.no[col - 1])

   col_names <- c(col_names, names(subset_data)[col])

   interval_numbers <- c(interval_numbers, interval_number) #Records the kinetic window

  }

  interval_number <- interval_number + 1 #Moves to the next kinetic window

 }

 result <- data.frame(cycle = interval_numbers, #Combines the data into a single data frame

           rep = col_names, der = -slopes * 60, #Expresses OCRs per minute

           condition=str_extract_part(col_names, "_", before = T)) #Extracts condition identifiers


 return(result) #Returns the results as a data frame for analysis or export

}
```

bioRxiv preprint doi: https://doi.org/10.1101/2025.05.16.654527; this version posted May 19, 2025. The copyright holder for this preprint (which was not certified by peer review) is the author/funder, who has granted bioRxiv a license to display the preprint in perpetuity. It is made available under aCC-BY 4.0 International license.

Supplementary tables

Table S1. Calibration constants for all media, solvent and drug treatments. P values represent the results of FDR-corrected Student's t tests relative to Milli-Q (MQ) H_2O controls. N=3-4.

Calibration constant	Solvent, media or drug treatment	I_R (mean ± SE)	P (≠ MQ H_2O)
k_0	MQ H_2O	95.97 ± 0.67	
	M9	99.25 ± 0.96	0.142
	0.2 % v/v DMSO	97.43 ± 0.47	0.204
	25 µM FCCP	97.74 ± 0.62	0.204
	24 mM sodium azide	99.03 ± 0.63	0.080
	25 µM FCCP + 24 mM sodium azide	97.98 ± 0.28	0.154
k_{100}	MQ H_2O	31.16 ± 0.18	
	M9	30.44 ± 0.43	0.776
	0.2 % v/v DMSO	30.98 ± 0.35	0.938
	25 µM FCCP	30.65 ± 0.24	0.725
	24 mM sodium azide	30.66 ± 0.28	0.776
	25 µM FCCP + 24 mM sodium azide	31.35 ± 0.14	0.938

bioRxiv preprint doi: https://doi.org/10.1101/2025.05.16.654527; this version posted May 19, 2025. The copyright holder for this preprint (which was not certified by peer review) is the author/funder, who has granted bioRxiv a license to display the preprint in perpetuity. It is made available under aCC-BY 4.0 International license.

Supplementary figures

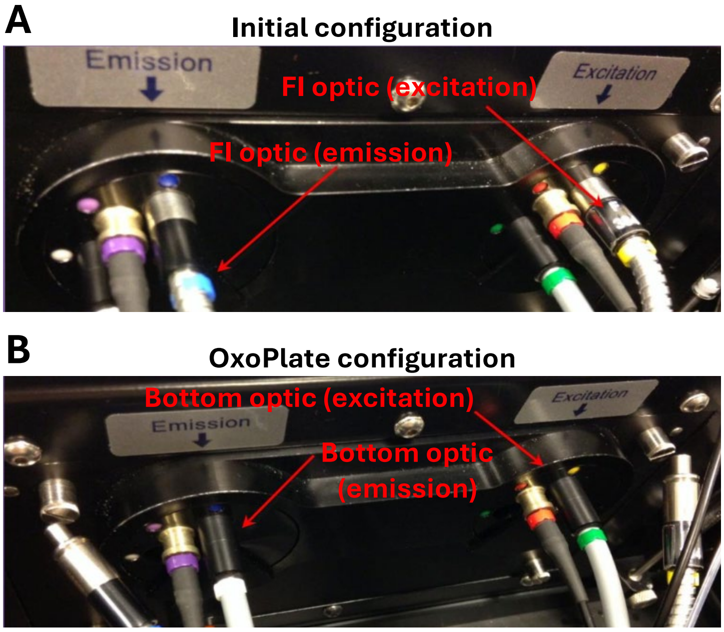

Figure S1. Light guide configuration for bottom-optic time-resolved fluorescence measurements using the BMG FLUOstar. A, Default light guide configuration with blue and yellow fluorescence intensity (FI) light guides. **B,** Corrected configuration in which the yellow and blue FI light guides have been replaced with the white and green bottom-optic light guides, leaving the FI light guides disconnected. Both Images were provided by PreSens.

bioRxiv preprint doi: https://doi.org/10.1101/2025.05.16.654527; this version posted May 19, 2025. The copyright holder for this preprint (which was not certified by peer review) is the author/funder, who has granted bioRxiv a license to display the preprint in perpetuity. It is made available under aCC-BY 4.0 International license.

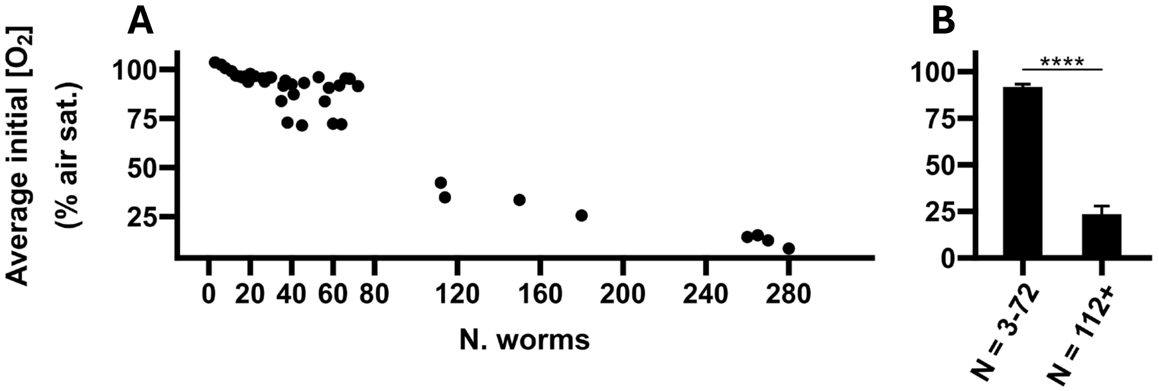

Figure S2. The impact of sample size on average starting [O_2]. A, Average first-measurement [O_2] in wells containing 3-280 animals. **B,** Pooled average first-measurement [O_2] over the linear OCR range (3-72 animals per well) compared to the non-linear range (112-280 animals per well). N = 40 wells. Annotations represent the results of ANOVA. ****, $P < 0.0001$.

bioRxiv preprint doi: https://doi.org/10.1101/2025.05.16.654527; this version posted May 19, 2025. The copyright holder for this preprint (which was not certified by peer review) is the author/funder, who has granted bioRxiv a license to display the preprint in perpetuity. It is made available under aCC-BY 4.0 International license.

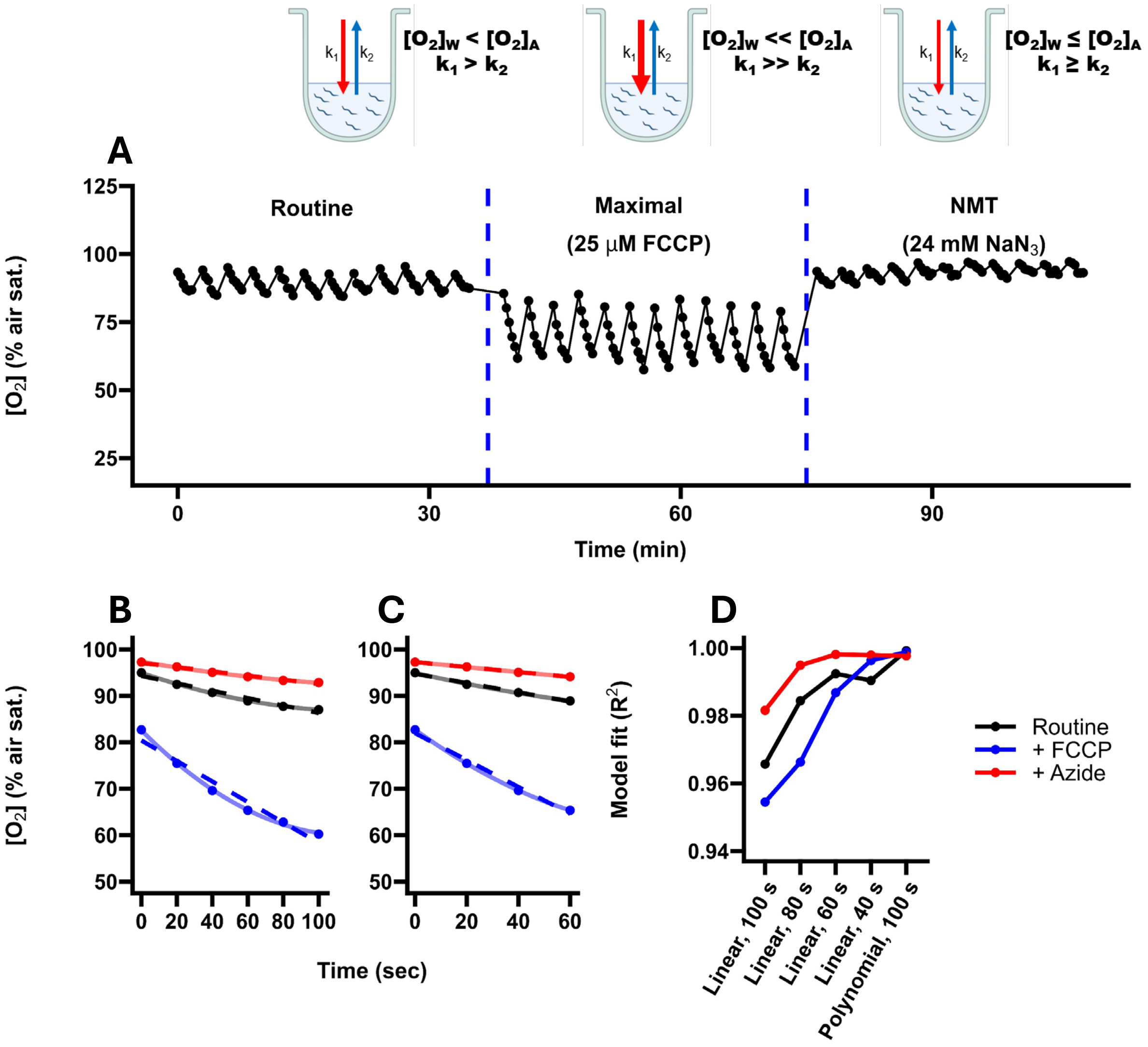

Figure S3. Defining optimal windows for the calculation of the *C. elegans* OCR. A, Simplified models of oxygen diffusion under periods of moderate (basal), high (maximal) and low (non-mitochondrial, NMT) oxygen consumption in uncovered OxoPlate wells with an accompanying average $[O_2]$ trace of *C. elegans* samples sequentially treated (dashed lines) with FCCP and sodium azide (NaN_3; re-produced from Figure 6; N = 15 wells). During periods of low oxygen consumption, the oxygen concentration within each well ($[O_2]_W$) is similar to the oxygen concentration of the ambient air ($[O_2]_A$), resulting in similar rates of oxygen diffusion (k_1 and k_2) between the environment and the contents of each well. In contrast, during periods of high oxygen consumption, the $[O_2]_W$ drops significantly below the $[O_2]_A$, causing a large net influx of oxygen into the contents of each well, masking the biological OCR. These models are simplified to only consider the contents of each well and the ambient air. However, the material of the OxoPlates likely acts as a separate oxygen reservoir and may need to be accounted for in an accurate model of oxygen diffusion. **B-C,** Oxygen concentrations averaged over each kinetic window displayed in panel A during basal, maximal and non-mitochondrial (NMT) respiration, with fitted curves of 2nd-order polynomials (solid lines) and linear regressions (dashed lines). Curves are fitted to the full 100 seconds in B, and the first 60 seconds in C. **D,** Goodness-of-fit (R^2) of linear models using subsets of the full measurement window (100 s) in 20 s increments, compared to the goodness-of-fit of 2nd-order polynomials fit to the full measurement window.

bioRxiv preprint doi: https://doi.org/10.1101/2025.05.16.654527; this version posted May 19, 2025. The copyright holder for this preprint (which was not certified by peer review) is the author/funder, who has granted bioRxiv a license to display the preprint in perpetuity. It is made available under aCC-BY 4.0 International license.

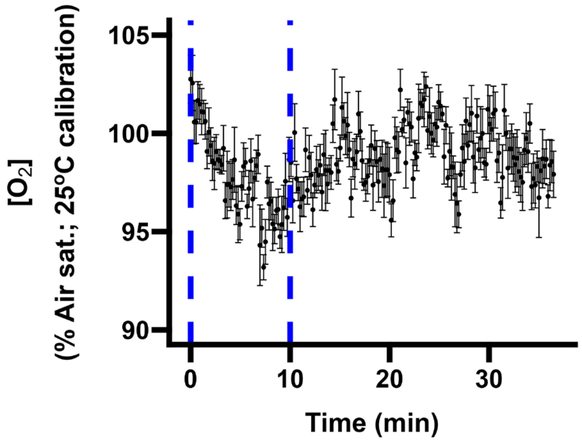

Figure S4. Estimate of temperature equilibration time following a shift from 20 °C to 25 °C. Temperature equilibration time was estimated based on the approximate length of time required for oxygen levels (quantified based on a two-point calibration at 25 °C) in solutions of Milli-Q to cease declining following transfer from 20 °C to 25 °C. N = 5 wells.

bioRxiv preprint doi: https://doi.org/10.1101/2025.05.16.654527; this version posted May 19, 2025. The copyright holder for this preprint (which was not certified by peer review) is the author/funder, who has granted bioRxiv a license to display the preprint in perpetuity. It is made available under aCC-BY 4.0 International license.

Time ID column

I_R value columns, replicates denoted by underscores

time	FCCP_1	FCCP_2	FCCP_3	DMSO_1	DMSO_2	DMSO_3	Control_1	Control_2	Control_3
0	32.17	32.8	32.27	32.37	32.84	33.38	31.4	31.63	32.41
20	32.2	33.28	32.95	32.81	34.79	34.51	31.78	32.93	33.24
40	33.26	34.67	33.51	33.83	34.57	34.9	32.84	34.24	34.27
60	33.76	34.72	34.34	34.42	35.32	35.15	33.69	34.43	35.01
80	34.16	34.35	33.71	35.24	35.62	34.83	33.39	34.45	34.82
100	33.35	34.73	34.25	34.59	36.71	35.32	33.41	34.15	34.49
179	31.52	33.42	32.14	31.69	32.87	32.75	30.92	31.98	31.96
199	32.66	32.8	32.12	32.54	34.67	33.37	31.91	31.95	32.97
219	32.87	33.69	33.63	33.31	35.17	33.09	32.2	33	33.84
239	33.65	34.77	33.69	34.46	36.29	34.46	32.64	34.54	34.1
259	33.82	35.13	34.54	35.28	36.23	36.17	32.82	34.69	34.62
279	34	35.38	34.11	34.79	37.67	35.97	33.89	34.8	34.59
358	31.46	32.69	31.98	31.37	32.36	32.16	30.19	31.27	32.42
378	31.75	33.13	32.92	32.62	33.71	33.06	31.17	31.86	33.52

....

....

....

Figure S5. Example data for automatic OCR analysis. The time identifier must be labelled "time" (in lowercase) and formatted in seconds. Each data column must be formatted with the name of the condition first (here FCCP, DMSO or Control) followed by a designation for the replicate (numbers or letters) separated by an underscore, i.e. FCCP_1 refers to the first replicate of the condition "FCCP".
